# Kôzulite, an Mn-rich alkali amphibole

**DOI:** 10.1107/S1600536810046015

**Published:** 2010-11-17

**Authors:** Madison C. Barkley, Hexiong Yang, Robert T. Downs

**Affiliations:** a Department of Geosciences, University of Arizona, 1040 E. 4th Street, Tucson, AZ 85721-0077, USA

## Abstract

The crystal structure of kôzulite, an Mn-rich alkali amphibole with the ideal formula NaNa_2_[Mn_4_^2+^(Fe^3+^,Al)]Si_8_O_22_(OH)_2_, tris­odium tetra­manganese iron/aluminium octa­silicate dihydroxide, was refined from a natural specimen with composition (K_0.20_Na_0.80_)(Na_1.60_Ca_0.18_Mn^2+^_0.22_)(Mn^2+^_2.14_Mn^3+^_0.25_Mg_2.20_Fe^3+^_0.27_Al_0.14_)(Si_7.92_Al_0.06_Ti_0.02_)O_22_[(OH)_1.86_F_0.14_]. The site occupancies determined from the refinements are *M*1 = 0.453 (1) Mn + 0.547 (1) Mg, *M*2 = 0.766 (1) Mn + 0.234 (1) Mg, and *M*3 = 0.257 (1) Mn + 0.743 (1) Mg, where Mn and Mg represent (Mn+Fe) and (Mg+Al), respectively. The average *M*—O bond lengths are 2.064 (1), 2.139 (1), and 2.060 (1) Å for the *M*1, *M*2, and *M*3 sites, respectively, indicating the preference of large Mn^2+^ for the *M*2 site. Four partially occupied amphibole *A* sites were revealed from the refinement, with *A*(*m*) = 0.101 (4) K, *A*(*m*)′ = 0.187 (14) Na, *A*(2) = 0.073 (6) Na, and *A*(1) = 0.056 (18) Na, in accord with the result derived from microprobe analysis (0.20 K + 0.80 Na), considering experimental uncertainties.

## Related literature

For more information on the geologic occurrence of kôzulite, see: Ashley (1986 ▶); Banno (1997 ▶); Hirtopanu (2006 ▶); Kawachi & Coombs (1993 ▶); Matsubara *et al.* (2002 ▶); Nambu *et al.* (1969 ▶, 1970 ▶, 1981 ▶); Watanabe *et al.* (1976 ▶). For the initial structural refinement of kôzulite, see: Fleischer & Nickel (1970 ▶); Kitamura & Morimoto (1972 ▶). For general background to the amphibole group, see: Hawthorne (1983 ▶); Hawthorne *et al.* (1995 ▶, 1996 ▶); Hawthorne & Harlow (2008 ▶). For background information on the amphibole group and nomenclature, see: Leake (1978 ▶); Leake *et al.* (1997 ▶, 2003 ▶); Mogessie *et al.* (2004 ▶).

## Experimental

### 

#### Crystal data

Na_3_[Mn_4_(FeAl)]Si_8_O_22_(OH)_2_
*M* *_r_* = 897.29
Monoclinic, 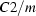

*a* = 9.9024 (7) Å
*b* = 18.1117 (12) Å
*c* = 5.2992 (4) Å
β = 104.034 (4)°
*V* = 922.04 (11) Å^3^
*Z* = 2
Mo *K*α radiation
μ = 2.87 mm^−1^
*T* = 293 K
0.06 × 0.05 × 0.04 mm

#### Data collection

Bruker APEXII CCD area-detector diffractometer
Absorption correction: multi-scan (*SADABS*; Sheldrick, 2008*a* ▶) *T*_min_ = 0.847, *T*_max_ = 0.894
7829 measured reflections
1977 independent reflections
1656 reflections with *I* > 2σ(*I*)
*R*_int_ = 0.019

#### Refinement

*R*[*F* ^2^ > 2σ(*F* ^2^)] = 0.023
*wR*(*F* ^2^) = 0.067
*S* = 1.08
1977 reflections
111 parameters
1 restraint
H-atom parameters not refined
Δρ_max_ = 0.58 e Å^−3^
Δρ_min_ = −0.59 e Å^−3^

### 

Data collection: *APEX2* (Bruker, 2003 ▶); cell refinement: *SAINT* (Bruker, 2005 ▶); data reduction: *SAINT*; program(s) used to solve structure: *SHELXS97* (Sheldrick, 2008*b* ▶); program(s) used to refine structure: *SHELXL97* (Sheldrick, 2008*b* ▶); molecular graphics: *XtalDraw* (Downs & Hall-Wallace, 2003 ▶); software used to prepare material for publication: *SHELXTL* (Sheldrick, 2008*b* ▶).

## Supplementary Material

Crystal structure: contains datablocks I, global. DOI: 10.1107/S1600536810046015/pk2265sup1.cif

Structure factors: contains datablocks I. DOI: 10.1107/S1600536810046015/pk2265Isup2.hkl

Additional supplementary materials:  crystallographic information; 3D view; checkCIF report

## Figures and Tables

**Fig. 1.**
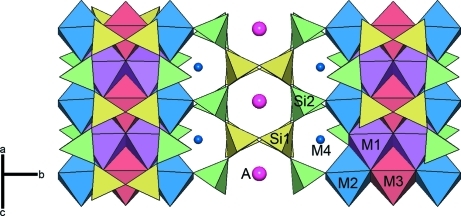
The crystal structure of kôzulite. Green and yellow tetrahedra represent [Si1O4] and [Si2O4] groups, respectively. Purple, blue, and red octahedra represent the M1, M2, and M3 octahedra, respectively. The small blue and large pink spheres represent the M4 and A cations, respectively.

## supplementary crystallographic information

### Comment

Kôzulite, with the ideal formula NaNa_2_[Mn_4_^2+^(Fe^3+^,Al)]Si_8_O_22_(OH)_2_, is an Mn-rich alkali member of the rock-forming amphibole family and was first described by Nambu *et al.* (1969). Kitamura and Morimoto (1972), in a meeting abstract, presented the structure refinement of a kôzulite crystal with the composition (Na_2.54_K_0.27_Ca_0.19_)(Mn_3.69_Mg_0.63_Fe^3+^_0.33_Al^3+^_0.31_)\ Si_8.00_O_21.78_[(OH)_2.18_F_0.04_]. However, they did not report its detailed structure information, such as atomic coordinates and displacement parameters. This study presents the first reported structure of kôzulite based on single-crystal X-ray diffraction data, as a part of our effort to build an integrated, web-based database of Raman spectra, X-ray diffraction, and chemistry data for all minerals (http://rruff.info).

The site occupancies determined from the refinements are *M*1 = 0.453 (1) Mn + 0.547 (1) Mg, *M*2 = 0.766 (1) Mn + 0.234 (1) Mg, and *M*3 = 0.257 (1) Mn + 0.743 (1) Mg, where Mn and Mg represent (Mn +Fe) and (Mg + Al), respectively. There results should be compared to those given by Kitamura and Morimoto (1972) for their kôzulite crystal: *M*1 = 0.78 Mn + 0.22 Mg, *M*2 = 0.95 Mn + 0.05 Mg, and *M*3 = 0.58 Mn + 0.42 Mg. The average M—O bond lengths are 2.064 (1), 2.139 (1), and 2.060 (1) Å for the *M*1, *M*2, and *M*3 sites, respectively. These values indicate that the *M*2 site is dominantly occupied by larger Mn^2+^, whereas the *M*1 and *M*3 sites should have similar amounts of (Mn^3+^ + Fe^3+^) and Mg. The relatively short average *M*3—O distance (*versus*. *M*1—O) suggests that Al^3+ ^is preferentially ordered into the *M*3 site. Our results on kôzulite are very similar to those observed by Hawthorne *et al.* (1995) for ungarettiite with the composition (K_0.15_Na_0.82_)(Na_1.97_Ca_0.03_)\ (Mn^2+^_1.66_Mn^3+^_2.97_Mg_0.34_Fe^3+^_0.03_Zn_0.01_)(Si_7.99_Al_0.01_)\ O_22_O_2._ The average M—O distances in ungarettiite are 2.03, 2.17, and 2.01 Å for the *M*1, *M*2, and *M*3 sites, respectively, pointing to the strong ordering of larger Mn^2+^ into the *M*2 site and smaller Mn^3+^ into *M*1 and *M*3.

Four partially occupied amphibole A sites [A(*m*), A(*m*)', A(2), and A(1)] were revealed from the structure refinements. The refinement shows that K prefers the A(*m*) site, whereas Na is distributed among the other three sites. The refined A site occupancies are 0.208 K + 0.764Na [A(*m*) = 0.208 (4) K, A(*m*)' = 0.374 (14) Na, A(2) = 0.146 (6)Na, and A(1) = 0.224 (18) Na], consistent with the result derived from microprobe analysis (0.20 K + 0.80Na), considering experimental uncertainties. The presence of two distinct A sites on the mirror plane, A(*m*) and A(*m*)' has also been observed in many other alkali amphiboles (*e.g.*, Hawthorne *et al.* 1996; Hawthorne and Harlow 2008).

### Experimental

The kôzulite specimen used in this study is from the type locality Tanohata Mine, Iwate Prefecture, Tohoku Region, Honshu Island, Japan and is in the collection of the RRUFF project (deposition No. R070122; http://rruff.info). The crystal chemistry was determined with a CAMECA SX50 electron microprobe (http://rruff.info) on the same single-crystal used for the collection of X-ray intensity data. The average composition (10 point analyses) yielded a chemical formula (normalized on the basis of 23 oxygen): (K_0.20_Na_0.80_)(Na_1.60_Ca_0.18_Mn^2+^_0.22_)˘Mn^2+^_2.14_Mn^3+^_0.25_Mg_2.20_Fe^3+^_0.27_Al_0.14_)˘Si_7.92_Al_0.06_Ti_0.02_)O_22_[(OH)_1.86_F_0.14_].

### Refinement

The chemical analysis and crystal-chemical considerations show that the C-group cations consist of Mn^2+^, Mn^3+^, Fe^3+^, Mg, and Al^3+^. Because of similar X-ray scattering powers, Fe and Mn were grouped together (represented by the scattering factor for Mn) and Mg and Al together (represented by Mg) throughout the structure refinements. No refinement was made for the cations in the M4 site (= Na_0.80_Ca_0.09_Mn^2+^_0.11_); they were assigned based on crystal-chemical considerations and previous studies on amphiboles (Hawthorne 1983). The total Mn and Mg in the *M*1 + *M*2 + *M*3 sites were fixed to those from the chemical analysis. To dampen the extreme correlations that would otherwise occur among the refined A-site variables, the isotropic displacement factors of these A sites were constrained to be equal (Hawthorne & Harlow 2008).

### Crystal data

**Table:**

Na_3_[Mn_4_(Fe)]Si_8_O_22_(OH)_2_	*F*(000) = 954
*M**_r_* = 897.29	*D*_x_ = 3.232 Mg m^−^^3^
Monoclinic, *C*2/*m*	Mo *K*α radiation, λ = 0.71073 Å
Hall symbol: -C 2y	Cell parameters from 2537 reflections
*a* = 9.9024 (7) Å	θ = 4.0–34.7°
*b* = 18.1117 (12) Å	µ = 2.87 mm^−^^1^
*c* = 5.2992 (4) Å	*T* = 293 K
β = 104.034 (4)°	Euhedral, brown
*V* = 922.04 (11) Å^3^	0.06 × 0.05 × 0.04 mm
*Z* = 2	

### Data collection

**Table:**

Bruker APEXII CCD area-detector diffractometer	1977 independent reflections
Radiation source: fine-focus sealed tube	1656 reflections with *I* > 2σ(*I*)
graphite	*R*_int_ = 0.019
φ and ω scans	θ_max_ = 34.4°, θ_min_ = 2.4°
Absorption correction: multi-scan (*SADABS*; Sheldrick, 2008a)	*h* = −15→15
*T*_min_ = 0.847, *T*_max_ = 0.894	*k* = −28→28
7829 measured reflections	*l* = −8→7

### Refinement

**Table:**

Refinement on *F*^2^	Primary atom site location: structure-invariant direct methods
Least-squares matrix: full	Secondary atom site location: difference Fourier map
*R*[*F*^2^ > 2σ(*F*^2^)] = 0.023	H-atom parameters not refined
*wR*(*F*^2^) = 0.067	*w* = 1/[σ^2^(*F*_o_^2^) + (0.0302*P*)^2^ + 1.2008*P*] where *P* = (*F*_o_^2^ + 2*F*_c_^2^)/3
*S* = 1.08	(Δ/σ)_max_ < 0.001
1977 reflections	Δρ_max_ = 0.58 e Å^−^^3^
111 parameters	Δρ_min_ = −0.59 e Å^−^^3^
1 restraint	

### Special details

**Table:**

Geometry. All e.s.d.'s (except the e.s.d. in the dihedral angle between two l.s. planes) are estimated using the full covariance matrix. The cell e.s.d.'s are taken into account individually in the estimation of e.s.d.'s in distances, angles and torsion angles; correlations between e.s.d.'s in cell parameters are only used when they are defined by crystal symmetry. An approximate (isotropic) treatment of cell e.s.d.'s is used for estimating e.s.d.'s involving l.s. planes.
Refinement. Refinement of *F*^2^ against ALL reflections. The weighted *R*-factor *wR* and goodness of fit *S* are based on *F*^2^, conventional *R*-factors *R* are based on *F*, with *F* set to zero for negative *F*^2^. The threshold expression of *F*^2^ > σ(*F*^2^) is used only for calculating *R*-factors(gt) *etc*. and is not relevant to the choice of reflections for refinement. *R*-factors based on *F*^2^ are statistically about twice as large as those based on *F*, and *R*- factors based on ALL data will be even larger.

### Fractional atomic coordinates and isotropic or equivalent isotropic displacement parameters (Å^2^)

**Table:**

	*x*	*y*	*z*	*U*_iso_*/*U*_eq_	Occ. (<1)
*M*1	0.0000	0.08447 (2)	0.5000	0.00933 (11)	0.4529 (19)
*M*1A	0.0000	0.08447 (2)	0.5000	0.00933 (11)	0.5471 (19)
*M*2	0.0000	0.182241 (18)	0.0000	0.00935 (9)	0.766 (2)
*M*2A	0.0000	0.182241 (18)	0.0000	0.00935 (9)	0.234 (2)
*M*3	0.0000	0.0000	0.0000	0.00682 (19)	0.256 (4)
*M*3A	0.0000	0.0000	0.0000	0.00682 (19)	0.744 (4)
M4A	0.0000	0.27152 (4)	0.5000	0.02191 (15)	0.79
M4B	0.0000	0.27152 (4)	0.5000	0.02191 (15)	0.09
M4C	0.0000	0.27152 (4)	0.5000	0.02191 (15)	0.11
Si1	0.28233 (4)	0.084285 (19)	0.28706 (7)	0.00858 (8)	
Si2	0.28734 (4)	0.16909 (2)	0.79054 (7)	0.00908 (8)	
O1	0.11555 (10)	0.08452 (5)	0.2127 (2)	0.01077 (18)	
O2	0.11918 (11)	0.16575 (6)	0.7170 (2)	0.01460 (19)	
O3	0.10330 (15)	0.0000	0.7118 (3)	0.0131 (3)	
O4	0.35819 (12)	0.24732 (6)	0.7901 (2)	0.0179 (2)	
O5	0.34710 (10)	0.12714 (6)	0.0742 (2)	0.01335 (19)	
O6	0.34473 (10)	0.11704 (6)	0.57692 (19)	0.01358 (19)	
O7	0.34279 (16)	0.0000	0.2884 (3)	0.0154 (3)	
AM	0.5211 (9)	0.0000	0.053 (2)	0.0149 (9)*	0.104 (4)
AM'	0.5562 (10)	0.0000	0.1298 (17)	0.0149 (9)*	0.187 (11)
A2	0.5000	−0.0215 (15)	0.0000	0.0149 (9)*	0.073 (6)
A1	0.5386 (18)	−0.0192 (18)	0.103 (4)	0.0149 (9)*	0.056 (6)

### Atomic displacement parameters (Å^2^)

**Table:**

	*U*^11^	*U*^22^	*U*^33^	*U*^12^	*U*^13^	*U*^23^
*M*1	0.00812 (19)	0.01278 (19)	0.00693 (19)	0.000	0.00151 (14)	0.000
*M*1A	0.00812 (19)	0.01278 (19)	0.00693 (19)	0.000	0.00151 (14)	0.000
*M*2	0.00981 (15)	0.00801 (14)	0.01081 (16)	0.000	0.00360 (11)	0.000
*M*2A	0.00981 (15)	0.00801 (14)	0.01081 (16)	0.000	0.00360 (11)	0.000
*M*3	0.0078 (3)	0.0059 (3)	0.0063 (3)	0.000	0.0009 (2)	0.000
*M*3A	0.0078 (3)	0.0059 (3)	0.0063 (3)	0.000	0.0009 (2)	0.000
M4A	0.0226 (4)	0.0251 (4)	0.0224 (4)	0.000	0.0139 (3)	0.000
M4B	0.0226 (4)	0.0251 (4)	0.0224 (4)	0.000	0.0139 (3)	0.000
M4C	0.0226 (4)	0.0251 (4)	0.0224 (4)	0.000	0.0139 (3)	0.000
Si1	0.00867 (16)	0.00776 (15)	0.00854 (16)	−0.00068 (11)	0.00063 (12)	0.00007 (11)
Si2	0.00895 (16)	0.00906 (15)	0.00918 (16)	−0.00125 (11)	0.00213 (12)	0.00087 (11)
O1	0.0088 (4)	0.0112 (4)	0.0115 (4)	−0.0013 (3)	0.0009 (3)	−0.0005 (3)
O2	0.0093 (4)	0.0202 (5)	0.0138 (5)	0.0015 (4)	0.0018 (3)	0.0031 (4)
O3	0.0139 (6)	0.0114 (6)	0.0133 (6)	0.000	0.0021 (5)	0.000
O4	0.0236 (5)	0.0115 (4)	0.0186 (5)	−0.0056 (4)	0.0054 (4)	0.0012 (4)
O5	0.0112 (4)	0.0171 (5)	0.0114 (4)	0.0000 (3)	0.0021 (3)	0.0049 (3)
O6	0.0115 (4)	0.0184 (5)	0.0099 (4)	0.0004 (3)	0.0006 (3)	−0.0039 (3)
O7	0.0149 (7)	0.0076 (5)	0.0227 (7)	0.000	0.0022 (5)	0.000

### Geometric parameters (Å, °)

**Table:**

*M*1—O3^i^	2.0223 (10)	M4A—O5^ix^	3.0160 (12)
*M*1—O3	2.0223 (10)	Si1—O1	1.6022 (11)
*M*1—O2	2.0551 (11)	Si1—O6	1.6226 (11)
*M*1—O2^ii^	2.0551 (11)	Si1—O5	1.6240 (10)
*M*1—O1	2.1148 (10)	Si1—O7	1.6391 (7)
*M*1—O1^ii^	2.1148 (10)	Si2—O4	1.5814 (11)
*M*2—O4^iii^	2.0197 (11)	Si2—O2	1.6166 (11)
*M*2—O4^iv^	2.0197 (11)	Si2—O5^x^	1.6598 (11)
*M*2—O2^v^	2.1425 (11)	Si2—O6	1.6748 (11)
*M*2—O2^ii^	2.1425 (11)	AM—O7^xi^	2.506 (7)
*M*2—O1^vi^	2.2554 (10)	AM—O5^xii^	2.809 (3)
*M*2—O1	2.2554 (10)	AM—O5^xi^	2.809 (3)
*M*3—O3^i^	2.0339 (15)	AM—O5^viii^	2.894 (4)
*M*3—O3^v^	2.0339 (15)	AM'—O7^xi^	2.643 (7)
*M*3—O1	2.0730 (10)	AM'—O6^xiii^	2.671 (6)
*M*3—O1^vi^	2.0730 (10)	AM'—O6^xiv^	2.671 (6)
*M*3—O1^vii^	2.0730 (10)	AM'—O5^xii^	2.809 (4)
*M*3—O1^viii^	2.0730 (10)	A2—O7^xi^	2.463 (4)
M4A—O4^ix^	2.3450 (12)	A2—O5^viii^	2.53 (2)
M4A—O4^iii^	2.3450 (12)	A2—O5^xi^	2.53 (2)
M4A—O2^ii^	2.3930 (13)	A1—O6^xiv^	2.53 (3)
M4A—O2	2.3930 (13)	A1—O5^xi^	2.55 (3)
M4A—O6^iii^	2.6283 (12)	A1—O7^xi^	2.644 (17)
M4A—O6^ix^	2.6283 (12)	A1—O5^viii^	2.70 (3)
M4A—O5^iii^	3.0160 (12)		
			
O3^i^—*M*1—O3	81.68 (6)	O1^vi^—*M*2—O1	76.60 (5)
O3^i^—*M*1—O2	175.50 (5)	O3^i^—*M*3—O3^v^	180.00 (6)
O3—*M*1—O2	94.98 (4)	O3^i^—*M*3—O1	84.47 (4)
O3^i^—*M*1—O2^ii^	94.98 (4)	O3^v^—*M*3—O1	95.53 (4)
O3—*M*1—O2^ii^	175.50 (5)	O3^i^—*M*3—O1^vi^	95.53 (4)
O2—*M*1—O2^ii^	88.50 (6)	O3^v^—*M*3—O1^vi^	84.47 (4)
O3^i^—*M*1—O1	83.68 (5)	O1—*M*3—O1^vi^	84.81 (5)
O3—*M*1—O1	96.35 (5)	O3^i^—*M*3—O1^vii^	95.53 (4)
O2—*M*1—O1	93.72 (4)	O3^v^—*M*3—O1^vii^	84.47 (4)
O2^ii^—*M*1—O1	86.24 (4)	O1—*M*3—O1^vii^	180.00 (6)
O3^i^—*M*1—O1^ii^	96.35 (5)	O1^vi^—*M*3—O1^vii^	95.19 (5)
O3—*M*1—O1^ii^	83.68 (5)	O3^i^—*M*3—O1^viii^	84.47 (4)
O2—*M*1—O1^ii^	86.24 (4)	O3^v^—*M*3—O1^viii^	95.53 (4)
O2^ii^—*M*1—O1^ii^	93.72 (4)	O1—*M*3—O1^viii^	95.19 (5)
O1—*M*1—O1^ii^	179.95 (6)	O1^vi^—*M*3—O1^viii^	180.00 (3)
O4^iii^—*M*2—O4^iv^	101.65 (7)	O1^vii^—*M*3—O1^viii^	84.81 (5)
O4^iii^—*M*2—O2^v^	92.64 (4)	O1—Si1—O6	111.41 (5)
O4^iv^—*M*2—O2^v^	97.47 (4)	O1—Si1—O5	112.64 (5)
O4^iii^—*M*2—O2^ii^	97.47 (4)	O6—Si1—O5	111.04 (6)
O4^iv^—*M*2—O2^ii^	92.64 (4)	O1—Si1—O7	110.92 (6)
O2^v^—*M*2—O2^ii^	163.97 (6)	O6—Si1—O7	106.35 (7)
O4^iii^—*M*2—O1^vi^	166.32 (4)	O5—Si1—O7	104.05 (7)
O4^iv^—*M*2—O1^vi^	91.14 (4)	O4—Si2—O2	117.68 (6)
O2^v^—*M*2—O1^vi^	80.77 (4)	O4—Si2—O5^x^	110.52 (6)
O2^ii^—*M*2—O1^vi^	86.65 (4)	O2—Si2—O5^x^	108.75 (6)
O4^iii^—*M*2—O1	91.14 (4)	O4—Si2—O6	106.26 (6)
O4^iv^—*M*2—O1	166.32 (4)	O2—Si2—O6	108.33 (6)
O2^v^—*M*2—O1	86.65 (4)	O5^x^—Si2—O6	104.45 (6)
O2^ii^—*M*2—O1	80.77 (4)		

Symmetry codes: (i) −*x*, −*y*, −*z*+1; (ii) −*x*, *y*, −*z*+1; (iii) −*x*+1/2, −*y*+1/2, −*z*+1; (iv) *x*−1/2, −*y*+1/2, *z*−1; (v) *x*, *y*, *z*−1; (vi) −*x*, *y*, −*z*; (vii) −*x*, −*y*, −*z*; (viii) *x*, −*y*, *z*; (ix) *x*−1/2, −*y*+1/2, *z*; (x) *x*, *y*, *z*+1; (xi) −*x*+1, −*y*, −*z*; (xii) −*x*+1, *y*, −*z*; (xiii) −*x*+1, *y*, −*z*+1; (xiv) −*x*+1, −*y*, −*z*+1.
